# Analyzing Health of Forcibly Displaced Communities through an Integrated Ecological Lens

**DOI:** 10.4269/ajtmh.22-0624

**Published:** 2023-02-06

**Authors:** Maia C. Tarnas, Carly Ching, Joleah B. Lamb, Daniel M. Parker, Muhammad H. Zaman

**Affiliations:** ^1^ Department of Population Health and Disease Prevention, University of California, Irvine, California;; ^2^ Department of Biomedical Engineering, Boston University, Boston, Massachusetts;; ^3^ Department of Ecology & Evolutionary Biology, University of California, Irvine, California

## Abstract

Health care among forcibly displaced persons is frequently driven by siloed approaches. Aspects of the built environment, social factors, and the bidirectional relationship between the changing ecosystem and residents are often ignored in health policy design and implementation. While recognizing factors that create a preference for siloed approaches and appreciating the work of humanitarian agencies, we argue for a new data-driven and holistic approach to understand the health of the forcibly displaced. It should be rooted in the realities of the emergence of new diseases, dynamic demographics, and degrading environments around the displaced communities. Such an approach envisions refugee and internally displaced camps as dynamic, complex ecosystems that alter, and are altered by, spatial and temporal factors. At the root of this approach is the necessity to work across disciplines, to think holistically, to go beyond treating single ailments, and to develop ethical approaches that provide dignity to those who are forcibly displaced.

The lives of millions of displaced individuals (including refugees, internally displaced persons, and stateless individuals) are characterized by exclusion, xenophobia, and global apathy. Displaced persons may live in formal or informal camps, or in other settings with few resources and unreliable access to essential commodities and services. Forced displacement, both in the short term and protracted, therefore has complex impacts on health.^1^^,^^2^

Factors that affect the health of forcibly displaced individuals can be split broadly into two realms: the physical and the social. Displaced communities, both during migration and when they are not moving, are often in locations that have limited access to essential health services. The geographic locations also often increase exposure to toxic and unstable environments, which can have immediate and chronic health impacts. The social attributes of displacement include legal status, nationality, and other socioeconomic factors that can likewise influence access to health services (and health itself), and force communities to live in a state of fear and anxiety. They may also affect the geographic settings in which displaced persons reside.

As humanitarian crises grow around the world and new humanitarian crises appear in South Asia, the Americas, Africa, and Europe, so do the camps that are sometimes home to displaced individuals for decades.^3^^–^^5^ In many camps across the globe, there are multiple generations that have been born and spent their entire lives in camps. The populations of these camps are neither fixed nor static, which can strongly influence epidemiological dynamics.^6^^–^^9^ Intergenerational factors (arrivals versus those who are born in the camps) create heterogeneity that leads to environmental changes, including those at microbial scales, not excluding changes in the gut microbiome. Changes in demography and environment are rarely studied from an integrated ecological and/or evolutionary lens, and are rarely part of the displaced individuals’ health landscape. This absence of scholarship and research allows for the continuation of siloed approaches and creates blind spots in understanding disease dynamics, emergence of new health challenges, provision of long-term care, and inefficient delivery of health services. We propose a more integrated approach to understand health challenges of the forcibly displaced—one that is multidisciplinary and multi-scale, and analyzes the communities and their environment from an ecological framework.

We define ecology as the interaction between organisms and their environment at multiple spatial scales. This includes interactions among microbes; among humans, disease vectors, and microbes; and between the physical landscape and humans, disease vectors, and microbes; and does not exclude noncommunicable diseases. Current approaches, even in the context of a One Health framework, can sometimes be siloed or at a single spatial or temporal scale. The common siloed approach through which many services are currently provided leads to inefficiencies in the provision of services, varying temporal coverage based on funding cycles and incomplete pictures of health. Environmental, ecological, and evolutionary considerations are often not even considered, posing further gaps in our understanding of the health of populations within such camps.

Though we have information on a higher, general level, we know few specifics about disease dynamics and the relationship of people and their environment in complex humanitarian emergencies.^10^ Siloed approaches neglect the reality that all components of humanitarian management have important health considerations when using an ecological lens. We therefore aim to understand the dynamic ecologies that connect vertically (i.e., from the microscale to the macroscale) and horizontally (between individuals, between environments, and between people and their environments) in forced displacement camps.

Understanding the ways that upstream factors can influence human health and microbial communities is useful in diagnosing and preventing poor health outcomes. Likewise, this type of big-picture lens is useful from a public health standpoint. A public health system that focuses on all health outcomes—communicable and noncommunicable, including mental health—and likewise considers the multidirectional health implications of human–biophysical environment interactions is useful for preventing disease. For those who are planning or administering camps, having a holistic view of the linkages between humans and their environments may lead to better planned and managed camps. Camp locations could often be better chosen, with health implications for the dwellers, for other communities in the same region, and for the environment. An approach that prioritizes local knowledge will lead to better understanding of the local ecological systems. To achieve this, we argue for considering displaced individual environs as adaptive ecosystems, with interactions at multiple temporal and spatial scales. An ecological lens challenges the siloed approach and allows for benefiting from a new understanding and new tools in the discipline. It helps us better understand the evolutionary and environmental pressures in the camps, and enables us to improve the lives of those who live in them.

## ENVIRONMENTAL AND SOCIAL ECOLOGIES

**Figure 1.**
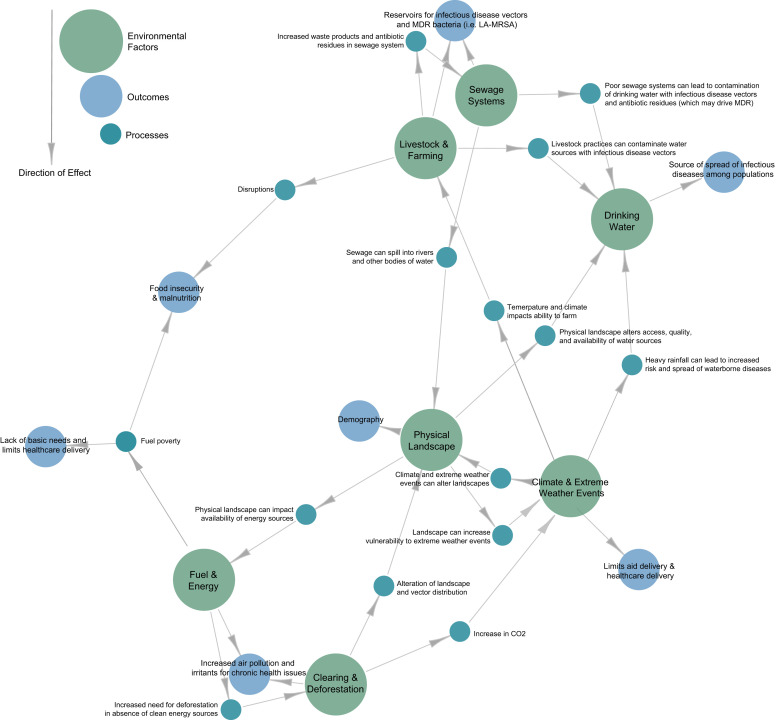
Noncomprehensive schematic of interactions between environmental factors and health within a displaced population camp. These interactions all occur within the context of broader phenomena, such as changing demography and climate change. Environmental factors are noted in green circles; outputs and outcomes are noted in blue circles. Interactions are represented directionally with gray lines and arrows, with expanded examples and process descriptions embedded outside teal circles. The network diagram was generated using the package igraph in R (version 4.1.2; R Foundation for Statistical Computing, Vienna, Austria). CO_2_ = carbon dioxide; LA-MRSA = livestock-associated–methicillin-resistant *Staphylococcus aureus*; MDR = multidrug resistant.

Humans, animals, and the environment influence each other in a codependent manner that varies with time and space, and is affected by both local and global factors. This multiscale dependence, although well recognized by those who are forced to live in the camps—has not been fully understood in research circles. To illustrate the importance of a multiscale dynamic ecological model in understanding the health of displaced individuals in camps, we offer Figure 1, which demonstrates and visualizes some of the ecologies and interactions occurring between the environment and health of displaced populations.

Social factors also play a critical role in the camp ecosystem and include the camp’s and individuals’ legal status, discrimination, mobility, employment, and host community relations. The camp’s legal status affects the provision and availability of services throughout the camp, as well as its development and care. The legal status of displaced individuals influences movement out of the camp, including being unable to seek external health care or obtain employment. Employment that is obtained may be in environments where certain diseases are more common. Differences in race, gender, legal status, and other demographic variables can lead to discrimination, inequality, and violence. These have direct and indirect effects on the health of displaced persons, and are compounded further by the individual’s vulnerable social and legal position, and lack of legal accountability for perpetrators. Studies have also found an association between gender inequality and environmental degradation.^11^ These social factors interact with the broader environment and work in tandem to influence health outcomes, including those related to mental health. Table 1 summarizes a sampling of these factors, though it is by no means an exhaustive discussion of these factors and interactions.

**Table 1:** Noncomprehensive list of environmental and social factors affecting displaced populations

Factor	Description
Built environment^12^	Infrastructure, including dwellings and water/sewage systems, that are poorly constructed and unsafe can increase exposure to toxic and unstable environments and disease.
Physical landscape^13^	The physical landscape informs disease ecology, affects resource availability, and increases vulnerability to weather events.
Camp location^12^^,^^14^^,^^15^	Camps are often located in suboptimal environments that would otherwise be sparsely inhabited and affect the provision of humanitarian aid. Camp creation leads to landscape changes to accommodate the population and built environment.
Fuel demand and supplies^13^^,^^16^^,^^17^	Availability of energy has impacts at the individual level, such as through the ability to cook or have electricity, and at the camp level through provision of health care and waste management. An insufficient energy supply often results in fuel poverty, increased deforestation, and malnutrition.^13^
Deforestation^13^	Deforestation changes the physical environment of the camps, including vector landscapes, often quite dramatically. It also contributes to climate change, and increased air pollution and irritants for chronic health issues.
Uncontrolled fires	The use of firewood has immediate dangers related to uncontrolled fires, which can spread rapidly in tightly packed camps.
Climate change and extreme weather events^18^^–^^20^	Temperature, climate, and extreme weather events can trigger displacement and affect individuals who have already been displaced.^18^ These events can also alter the physical landscape, exacerbate the spread of infectious diseases, and affect the provision of humanitarian aid.
Water and sewage systems^20^^,^^21^	Unsafe drinking water and stagnant contaminated water can be sources of infectious diseases and long-term health impacts related to the consumption of naturally derived compounds.^22^^–^^25^ Clean water can also affect the provision of health care.
Groundwater quality^22^^,^^26^^,^^27^	Insufficient sewage systems may introduce or reintroduce viruses and bacteria into groundwater and surface water, disrupting the natural ecosystem and contributing to disease spread.^22^^,^^27^ Land clearing and burning, food production, and development or land hardening also release pollutants into these water sources.
Livestock^28^^–^^30^	Livestock serve as reservoirs for diseases and can attract disease vectors and multidrug-resistant bacteria, especially if practices are unregulated. Animal waste can also contaminate the water supply.
Livelihood production^28^	Means of livelihood, especially those that rely on water-intensive agricultural practices or livestock, can affect water resources.^28^ Such means are also vulnerable to changes in temperature and climate.
Watershed health and viability^26^^,^^31^	Long-term viability of water sources depends on watersheds around camps, which are affected by the removal of native vegetation, changing hydrology, alteration of the microbial communities, and the addition or increase of pollutants in runoff.^31^
Camp legal status^32^	The legal status affects the provision and availability of services throughout the camp, as well as the camp’s development and care.
Political and conflict environment	Political and/or conflict-related challenges cause variation in camp services over time, especially when camps are in areas of strategic importance.
Discrimination, inequality, and violence^33^^–^^35^	Differences in race, gender, legal status, and other demographic variables affect how an individual is treated upon entering and living within the camp. Women and girls are particularly vulnerable to gender-based violence, and lack an established justice system and access to appropriate physical and/or mental care.^33^^–^^35^
Mobility^36^^,^^37^	Individuals may have their movement restricted or may be completely unable to leave a camp legally, which limits access to health services and their ability to seek employment or education.
Occupational opportunity^38^	Displaced persons are often limited with regard to occupational opportunities as a result of legal and structural barriers to accessing work. Because of this, many are forced to work in environments where certain diseases are more common or they may work illegally.
Host community relationships	Host community relationships may be strained by actions such as deforestation, unsustainable land use, and employment outside of camps. These relationships can have marked effects on the longevity of the camp.
Demography	Demography has impacts across all sectors. Changes in demography can affect disease burden, required services, livelihood, physical landscapes, fuel demands, and other important components of the camp ecosystem.

## DISCUSSION

The United Nations High Commissioner for Refugees views camps as “temporary solutions of last resort (page 2),” yet the formation of camps is so common that it warrants better planning.^39^ Although we fully acknowledge that displaced persons should not be confined to camps and strongly argue for dignified living conditions for those who have been forced to leave their homes, we argue that for those who are in camps, an expanded understanding of the interacting ecologies will help us improve lives as other policies that provide a dignified existence out of the camps are prioritized. We have thus far focused on situations in which displaced persons aggregate in camps, though many are instead living in urban environments. These environments likewise tend to be unsafe and unhealthy. An ecological lens allows us to understand more fully the living conditions in areas such as these, though other factors (including public health policy) are needed to address the needs of this specific population.^40^

We recognize that this lens has its limitations. Individual camps create unique contexts and ecosystems, meaning that ecological interactions may vary greatly among camps. The adoption of this approach may have numerous logistic barriers, though these may be largely a result of the siloed approach this lens attempts to deconstruct. Developing a better understanding of how different components of this ecosystem interact, including at different space-/timescales, could lead to better planning and interventions to improve the health of both populations and environments in these difficult settings.

## RECOMMENDATIONS

We need more partnerships that are cross-cutting between silos and that bring together humanitarian aid providers, camp planners, ecologists, public health professionals, health-care providers, environmental practitioners, lawyers, policymakers, and other experts of factors that contribute to the ecology of camps. Integrated policy needs to be developed based on assessing connections among systems so that evidence-based decisions can be made about how interventions may influence outcomes in multiple sectors. Sharing resources and taking an integrated approach will assist in buffering risk and creating more effective and proactive governance. We recommend research and collaboration that increases our understanding of interactions among camps, their environments, and broader ecosystems to incorporate this knowledge into future camps. This includes improving data gathering and analysis to incorporate local knowledge and partners with an emphasis on actively engaging women.^11^ We recommend having the data ecosystem be considered more seriously and prioritized in these settings.

Making decisions through an ecological lens does not need to preclude immediate aid provision. Through our recommended collaboration, we foresee a pathway to integrate ecological considerations into camp planning, development, management, and sustainment. However, we must first learn how we can adapt this lens in a way that is practical in real-time camp settings. As the first step in this process, we recommend a series of workshops that brings together different actors across silos to make concrete pathways for the inclusion of ecological considerations in camps. This piece is but the first contribution in what we anticipate, and indeed hope, will be much larger conversations around improving the care and services we provide displaced persons.
